# Scurvy: A Case Report and Literature Review

**DOI:** 10.7759/cureus.14312

**Published:** 2021-04-05

**Authors:** Jason M Thomas, Kathryn M Burtson

**Affiliations:** 1 Internal Medicine, Wright Patterson Air Force Base/Wright State University, Dayton, USA

**Keywords:** scurvy, ascorbic acid, hemodynamic instability, perifollicular hemorrhage

## Abstract

Scurvy is a rare disease which can manifest in a variety of presentations. Classically, scurvy is associated with poor dentition and bleeding diatheses. Rarely, scurvy can present with life-threatening hemodynamical instability.

Herein, we report the case of a 69-year-old female with a history of hypertension and depression who presented with four months of weakness and a 20-pound weight loss. Her presentation was complicated by lower extremity bruising and myalgias over the last three weeks. The patient’s blood pressure in the emergency department was 86/54 mmHg. On further examination, she had poor dentition and extensive ecchymoses in different stages of resolution over her posterior thighs and calves. The patient was also noted to have perifollicular hemorrhages. An ascorbic acid level was checked and the result was 0.0 mg/dL (normal range: 0.4 to 2.0 mg/dL). During her admission, she slowly improved with a provided diet and multivitamins. Her blood pressure consistently remained over 120/65 mmHg. The patient was advised to adjust her diet and take supplemental ascorbic acid. On a follow-up visit two weeks later, the patient endorsed an improvement in pain and exercise tolerance and was noted to have marked improvement in skin findings.

Ascorbic acid is an essential piece of multiple biochemical pathways. Humans are required to attain ascorbic acid from their diet. People who consume diets lacking in ascorbic acid develop scurvy.

## Introduction

Scurvy is a re-emerging disease of antiquity that can be debilitating and cause life-threatening hemodynamic instability. It is a devastating disease with a very simple cure and is prevalent even in resource-rich nations. In the 2003-2004 National Health and Nutrition Examination Survey, 7.1 ± 0.9% of the total population was found to have vitamin C deficiency [1]. Despite this prevalence, there are rare reports of manifest scurvy in the United States in the last 30 years. Nonspecific symptoms, such as irritability, fatigue, weight loss, and aching pains, are under-recognized as latent scurvy. An ascorbic acid level less than 10 mg/dL is associated with petechial hemorrhage, follicular hyperkeratosis, failure of wound healing, anemia, loosened teeth, and bleeding gums. In later stages of the disease, vasomotor instability and shock have been documented [2]. Clinicians must be aware of signs and symptoms of scurvy and the importance of ascorbic acid in hemodynamic stability. 

## Case presentation

**Figure 1:**
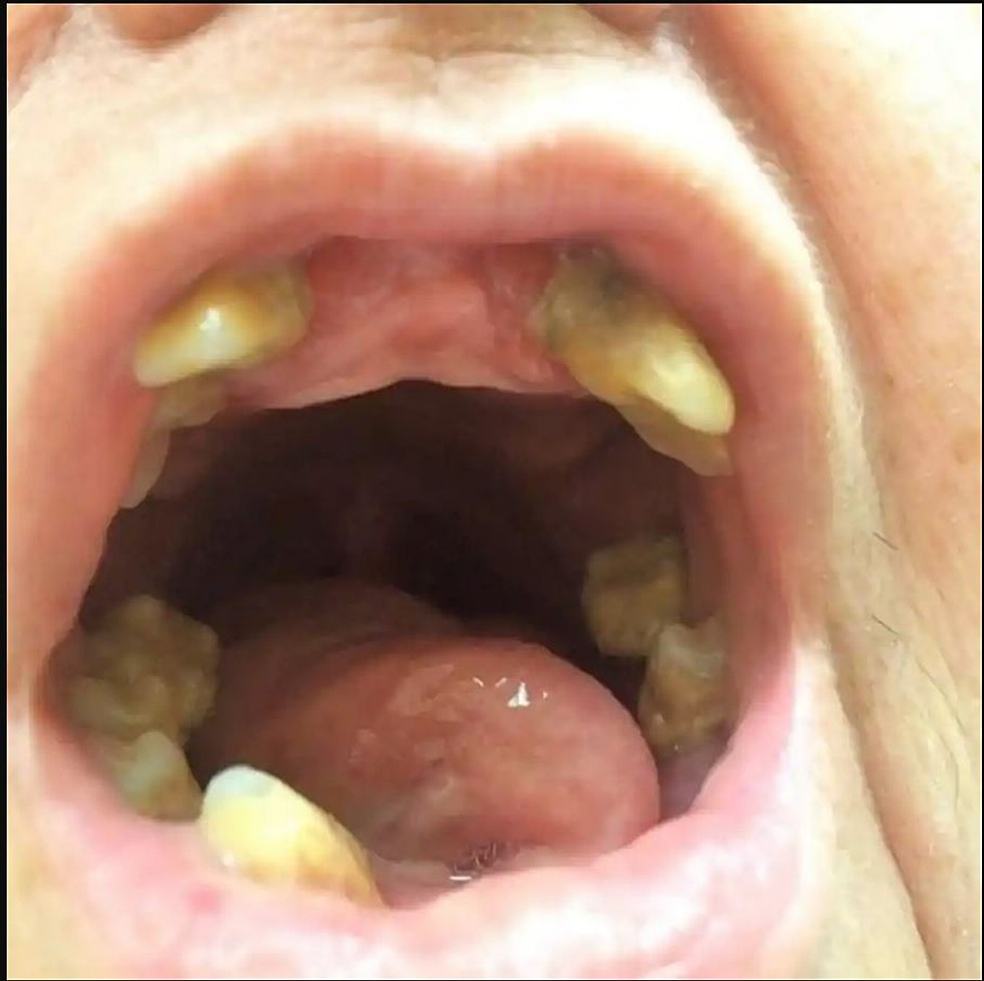
Poor dentition

**Figure 2:**
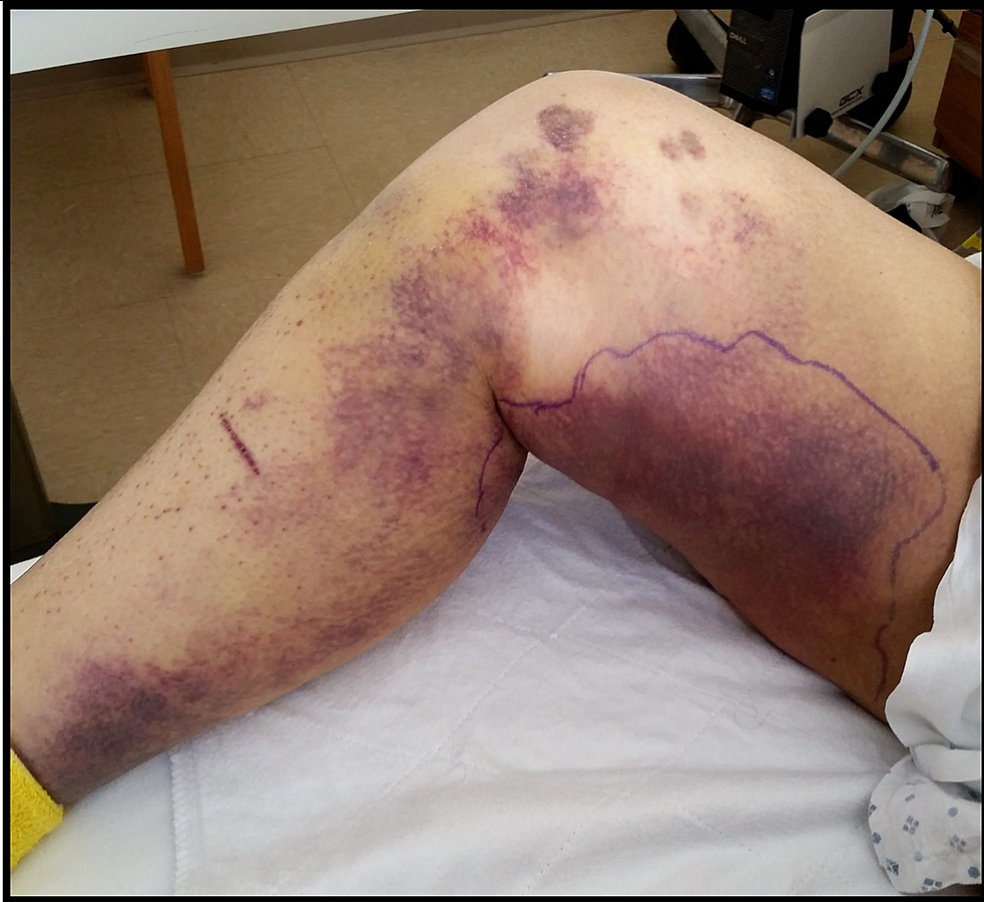
Extensive ecchymoses in different stages of resolution over posterior thighs and calves

A 69-year-old female with a history of hypertension and depression presented with a four-month history of weakness and a 20-pound weight loss. She had associated symptoms of lower extremity bruising and myalgias over the previous three weeks. She denied hematemesis, coffee-ground emesis, melena, hematochezia, fevers, or night sweats. The patient was taking 81 mg of aspirin, 320 mg of valsartan, 5 mg of amlodipine, and 100 mg of metoprolol succinate daily. She denied any relevant family history. The patient’s blood pressure in the emergency department was 86/54 mmHg with a temperature of 97°F, heart rate of 52, respiratory rate of 17, and a oxygen saturation of 99% on room air. On examination, she had poor dentition and extensive ecchymoses in different stages of resolution over her posterior thighs and calves (Figures 1-2). Laboratory studies revealed a hemoglobin of 9.5 g/dL, albumin of 2.3 g/dL, and erythrocyte sedimentation rate (ESR) of 75 mm/hr. The platelet count, international normalized ratio (INR), B12, folate, and thyroid-stimulating hormone (TSH) were within normal limits.

For the first two days of her admission, the patient continued to be hypotensive with systolic blood pressures ranging from 93 - 97 mmHg and diastolic blood pressures ranging from 45 - 54 mmHg. She had a transient response to resuscitation, requiring intensive care monitoring and four liters of crystalloid to maintain adequate perfusion. Her differential included gastrointestinal bleeding, myelodysplastic syndrome, amyloidosis, Factor XIII deficiency, platelet dysfunction, and scurvy. The bone marrow biopsy, serum protein electrophoresis (SPEP), light chains, platelet function analysis, and factor XIII levels were unrevealing. A fecal occult tested positive, and a subsequent esophagogastroduodenoscopy revealed antral ulcers.

With the suspicion of scurvy, a more detailed history was taken. She denied alcohol use. Her diet was limited to canned green beans, burgers, eggs, and bananas. On repeat examination, the patient was noted to have perifollicular hemorrhages. An ascorbic acid level was checked and resulted at 0.0 mg/dL (normal range: 0.4 to 2.0 mg/dL).

**Figure 3:**
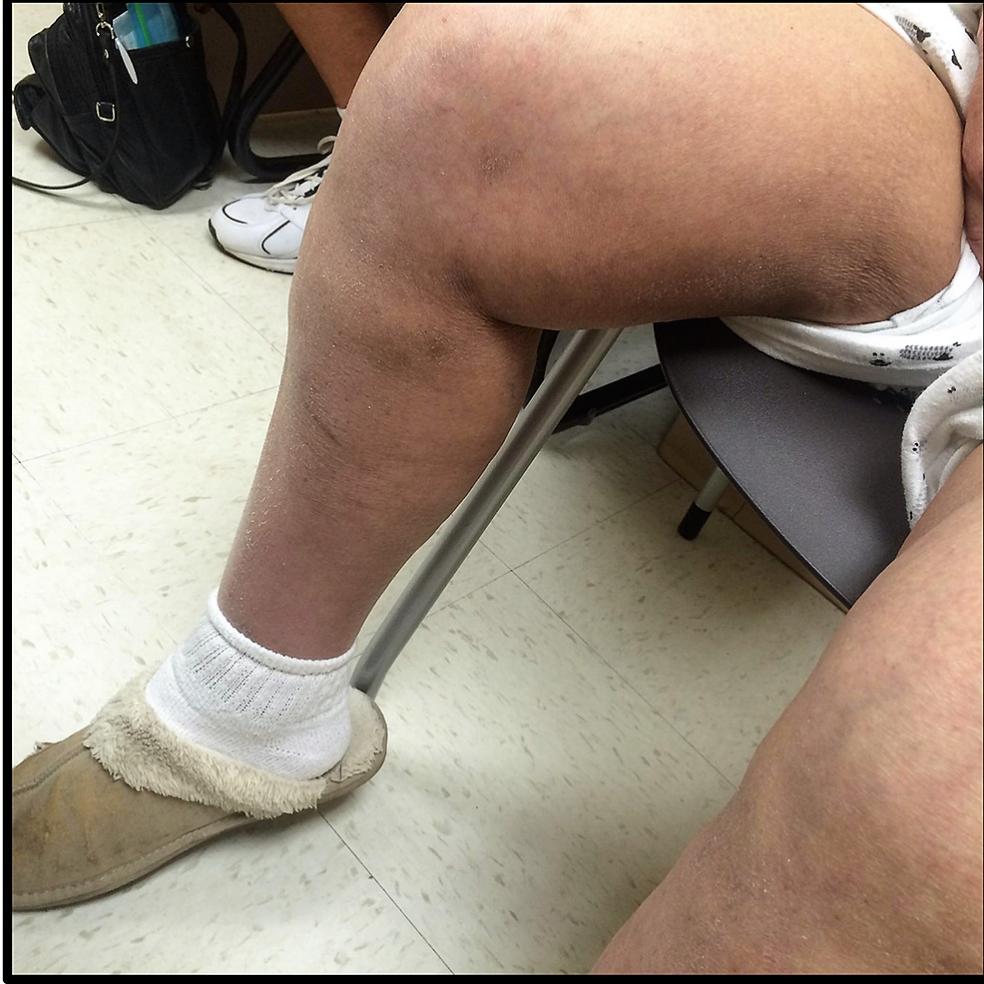
Marked improvement in skin findings

During her admission, the patient slowly improved with a provided diet and daily multivitamins. Her blood pressure consistently remained over 120/65 mmHg. The patient was advised to adjust her diet and take supplemental ascorbic acid. On a follow-up visit two weeks later, the patient endorsed an improvement in pain and exercise tolerance and was noted to have marked improvement in skin findings (Figure 3).

## Discussion

Ascorbic acid is an essential part of multiple biochemical pathways. While animals produce ascorbic acid from glucose, humans lack a functional copy of a crucial enzyme needed to complete the process [3]. Humans are required to attain ascorbic acid from their diet. Foods rich in vitamin C include tomatoes, potatoes, and citrus fruits. People who consume diets lacking ascorbic acid develop scurvy.

Frequently taught with historical relevance, 7% of the population in the United States suffers from scurvy [1]. Most individuals with this disease are alcoholics and homeless patients; however, literature exists of patients with scurvy caused by self-limited diets [4].

Due to the integral role of ascorbic acid in numerous biochemical pathways, scurvy presents with a variety of symptoms, including bleeding diathesis, anemia, fatigue, myalgias, perifollicular palpable purpura, corkscrew hairs, and gingival swelling [4-5]. Ascorbic acid is also crucial in the conversion of L-tyrosine to levodopa (L-​DOPA), a precursor for catecholamines. The biochemical synthesis of epinephrine requires ascorbic acid along two different stages in production. Whilst hypotensive, our patient did not mount a compensatory tachycardia. Although she was taking metoprolol succinate daily, this lack of compensation was also attributed to the disruption of her catecholamine synthesis (3). 

Rarely, patients can present with hemodynamic instability, even to the point of shock and requiring pressor support [2]. Fortunately, in cases of scurvy-related shock, the treatment is supplemental ascorbic acid. Patients will respond to therapy within 48 hours [4].

## Conclusions

Scurvy is a devastating disease with a very simple cure and is prevalent even in resource-rich nations. Clinicians must be aware of signs and symptoms of scurvy and the importance of ascorbic acid in hemodynamic stability.

## References

[1] Schleicher RL, Carroll MD, Ford ES, Lacher DA (2009). Serum vitamin C and the prevalence of vitamin C deficiency in the United States: 2003-2004 National Health and Nutrition Examination Survey (NHANES). Am J Clin Nutr.

[2] Zipursky JS, Alhashemi A, Juurlink D (2014). A rare presentation of an ancient disease: scurvy presenting as orthostatic hypotension. BMJ Case Rep.

[3] Francescone MA, Levitt J (2005). Scurvy masquerading as leukocytoclastic vasculitis: a case report and review of the literature. Cutis.

[4] Smith A, Di Primio G, Humphrey-Murto S (2011). Scurvy in the developed world. CMAJ.

[5] Velandia B, Centor RM, McConnell V, Shah M (2008). Scurvy is still present in developed countries. J Gen Intern Med.

